# Interaction between inflammatory mediators and miRNAs in *Helicobacter pylori* infection

**DOI:** 10.1111/cmi.12587

**Published:** 2016-04-14

**Authors:** Ana Flávia Teixeira Rossi, Aline Cristina Targa Cadamuro, Joice Matos Biselli‐Périco, Kátia Ramos Moreira Leite, Fábio Eduardo Severino, Patricia P. Reis, José Antonio Cordeiro, Ana Elizabete Silva

**Affiliations:** ^1^ UNESP, São Paulo State UniversityDepartment of BiologyRua Cristóvão Colombo, 2265São José do Rio PretoSPBrazil; ^2^ USP, São Paulo UniversityFaculty of Medicine, Department of SurgeryAvenida Dr. Arnaldo, 455São PauloSPBrazil; ^3^ UNESP, São Paulo State UniversityFaculty of Medicine, Department of Surgery and OrthopedicsAvenida Prof. MontenegroBotucatuSPBrazil

## Abstract

*Helicobacter pylori* cause chronic inflammation favouring gastric carcinogenesis, and its eradication may prevent malignant transformation. We evaluated whether *H. pylori* infection and its eradication modify the expression of inflammatory mediators in patients with chronic gastritis. Furthermore, we assessed whether microRNAs modulate inflammatory pathways induced by *H. pylori* and identified miRNA–gene interaction networks. mRNA and protein expression of *TNFA*, *IL6*, *IL1B*, *IL12A*, *IL2* and *TGFBRII* and miRNAs miR‐103a‐3p, miR‐181c‐5p, miR‐370‐3p, miR‐375 and miR‐223‐3p were evaluated in tissue samples from 20 patients with chronic gastritis *H. pylori* negative (Hp−) and 31 *H. pylori* positive (Hp+), before and three months after bacterium eradication therapy, in comparison with a pool of Hp− normal gastric mucosa. Our results showed that *H. pylori* infection leads to up‐regulation of *TNFA*, *IL6*, *IL12A* and *IL2* and down‐regulation of miRNAs. Bacterium eradication reduces the expression of *TNFA* and *IL6* and up‐regulates *TGFBRII* and all investigated miRNAs, except miR‐223‐3p. Moreover, transcriptional profiles of inflammatory mediators and miRNAs after eradication are different from the non‐infected group. Deregulated miRNA–mRNA interaction networks were observed in the Hp+ group before and after eradication. Therefore, miRNAs modulated cytokine expression in the presence of *H. pylori* and after its eradication, suggesting that miRNAs participate in the pathological process triggered by *H. pylori* in the gastric mucosa.

## Introduction

*Helicobacter pylori* (*H. pylori*) is a Gram‐negative and spiral‐shaped bacterium with great ability to colonize the human stomach (Salama *et al*., 2013). This pathogen triggers an inflammatory response in the gastric mucosa characterized by presence of polymorphonuclear cells and lymphocytes, besides the stimulation of nuclear factor (NF)‐κB, production of survival and proliferation factors and pro‐ and anti‐inflammatory cytokines (Cadamuro *et al*., 2014). Persistent infection promotes the chronic inflammation that causes mucosal damage and the consequent development of gastric lesions such as chronic gastritis, which can progress to gastric atrophy, intestinal metaplasia, dysplasia and gastric cancer (Kandulski *et al*., 2008). Thus, *H. pylori* has been classified as type I carcinogen (IARC, 1994).

Several virulence factors related to the pathogenicity of this bacterium help colonize the human stomach, such as Cytotoxin‐associated gene A (CagA) (Wang *et al*., 2014). The *cagA* gene is located in *cag* pathogenicity island (*cag*PAI) that also contains genes, which give rise to the bacterial type IV secretion system (T4SS) (Censini *et al*., 1996) responsible for injecting the CagA protein and other bacterial products into gastric epithelial cells (Backert *et al*., 2000). This oncoprotein exerts several effects on gastric cells resulting in cytoskeleton rearrangement, disruption of cell polarity and stimulation of mitogenic and pro‐apoptotic responses, as well as a greater degree of mucosal inflammation (Brandt *et al*., 2005).

Studies have shown that *H*. *pylori* infection deregulates host gene expression, such as receptors and co‐receptors involved in bacterial recognition, signal transduction, immune and inflammatory response mediators, apoptosis, proliferation and metabolism‐related genes in infected versus non‐infected individuals (Hofman *et al*., 2007; Yang *et al*., 2012; Cadamuro *et al*., 2015). As a consequence, gene expression changes can influence the intensity of the host response against infection.

In addition, gene expression is epigenetically modulated by microRNAs (miRNAs), which are small non‐coding RNA molecules that negatively regulate post‐transcriptional gene expression mainly through mRNA degradation or translational repression (Bartel, 2004). Considering that a single miRNA is able to target several mRNA molecules, deregulated miRNA expression may affect multiple signalling pathways contributing to the development and progression of inflammatory diseases and cancer (Ranjha and Paul, 2013). Furthermore, *H. pylori* can alter miRNA expression in gastric mucosa (Matsushima *et al*., 2011) and miRNAs may regulate inflammation through their action on target mRNAs encoding pro‐ and anti‐inflammatory cytokines that play a role on host defence response against this pathogen (Cadamuro *et al*., 2014).

*H. pylori* eradication results in significant regression of early gastric lesions but not in advanced lesions as intestinal metaplasia and dysplasia (Wang *et al*., 2011; Lee *et al*., 2013), indicating that there is a ‘point of no return’ in which genetic alterations are irreversible despite bacterium elimination (Wang *et al*., 2011). However, knowledge about changes in global genetic profiles and signalling pathways deregulated during *H. pylori* infection and after its eradication is still limited. Herein, we assessed whether *H. pylori* infection and its eradication, beyond its *cagA* virulence genotype, change the expression of inflammatory mediators (*TNFA*, *IL6*, *IL1B*, *IL12A*, *IL2* and *TGFBRII*) in chronic gastritis patients. Furthermore, we selected five miRNAs (miR‐103a‐3p, miR‐181c‐5p, miR‐370‐3p, miR‐375 and miR‐223‐3p) by public database as DIANA‐microT (Reczko *et al*., 2012; Paraskevopoulou *et al*., 2013) and TargetScan (Agarwal *et al*., 2015) involved with inflammatory response and gastric carcinogenesis not yet extensively studied on its association with *H. pylori* infection and to have cytokines genes as targets. In addition, we assessed whether these miRNAs have a role on mRNA deregulation in *H. pylori* positive versus *H. pylori* eradicated versus *H. pylori* negative gastric tissue samples, through the identification of miRNA–mRNA interaction networks. Our results show that *H. pylori* infection, regardless of *cagA* genotype, leads to up‐regulated expression of most inflammatory mediators in inflammatory and epithelial cells of gastric mucosa. *H. pylori* eradication partially reduces inflammatory response; however, the transcriptional patterns of this group are different from the non‐infected group. Furthermore, we identified miRNAs as modulators of inflammatory gene expression in *H. pylori* positive samples and after *H. pylori* eradication.

Therefore, miRNAs may participate in the pathological process triggered by *H. pylori* in the gastric mucosa, influencing the host inflammatory response against infection.

## Results

### Characterization of the inflammation scoring

In all cases (Hp+ and Hp− groups), the inflammatory infiltrate is mainly lymph mononuclear, predominantly composed by lymphocytes and sometimes plasma cells. Macrophages and neutrophils are rare and sparce.

The scoring of inflammation was classified in mild, moderate and severe based on the inflammatory infiltrate. In the Hp+ group before treatment, there was the predominance of severe inflammation (54%). After eradication, the scoring of inflammation was significantly reduced (*p* = 0.022) relative to Hp+ group, with 50% of cases classified as mild. The non‐infected group presented 47%, 33% and 20% of cases with inflammation degree mild, moderate and severe respectively.

### Molecular diagnosis for cagA and rate of bacterial *eradication*

Of the 31 Hp+ patients with chronic gastritis, 22 were genotyped for *cagA* using multiplex PCR, from which 45.4% (10/22) were *cagA*‐positive. Among the *cagA*‐positive and *cagA*‐negative samples, approximately 60% have eradicated the bacterium, showing that the presence of this virulence genotype did not influence the rate of bacterial eradication (*p* = 1.000). For further analyses, we excluded the 10 patients who have not eradicated the bacterium after treatment, thus enabling the analysis specifically of the influence of bacterium eradication on miRNA and gene expression.

### Expression of inflammatory mediator genes in H. pylori+ *versus* H. pylori *eradicated versus* H. pylori *negative patient samples*

**Figure 1:**
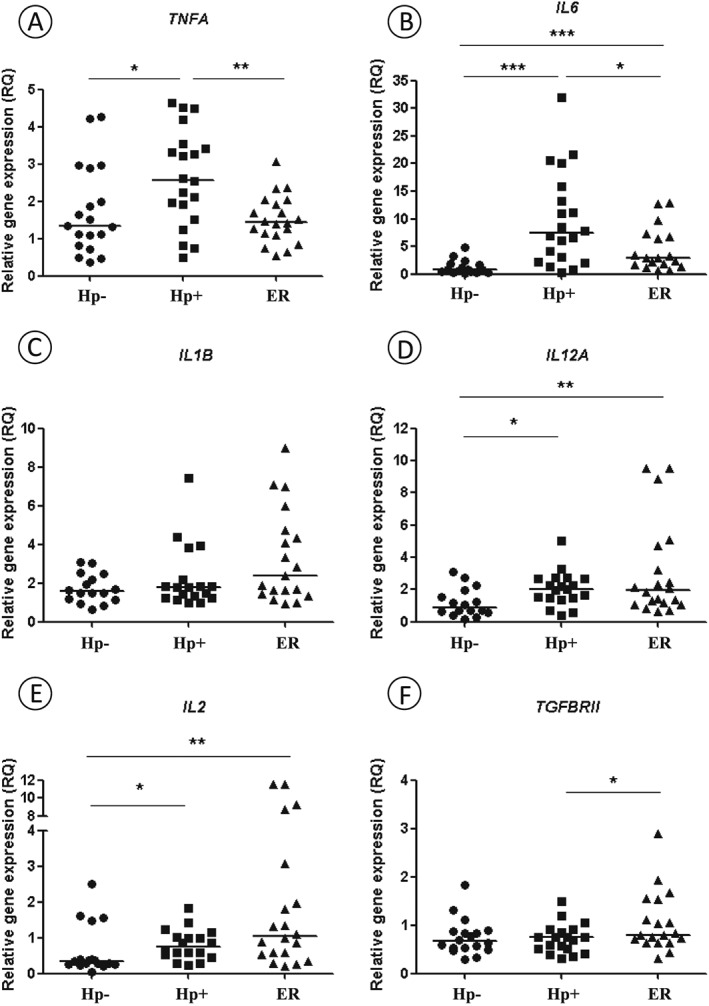
Relative expression of inflammatory mediators genes: *TNFA* (A), *IL6* (B), *IL1B* (C), *IL12A* (D), *IL2* (E) and *TGFBRII* (F) mRNA in patients with chronic gastritis *H. pylori*‐negative (Hp−) and *H. pylori*‐positive before (Hp+) and after eradication treatment (ER) groups. Data are presented as RQ values, and each point represents one individual. Line symbolizes RQ median for each group. * p ≤ 0.05; ** p ≤ 0.01; *** p ≤ 0.001.

mRNA expression levels of inflammatory mediator genes were examined in Hp+ samples before treatment, after *H. pylori* eradication and in Hp− patient groups using quantitative TaqMan® PCR. After normalization with the reference genes and a pool of Hp− normal gastric mucosa, statistically significantly up‐regulated mRNA expression levels of *TNFA*, *IL6*, *IL12A* and *IL2* were detected in the Hp+ group before treatment compared to Hp− group (*p* = 0.024, <0.001, 0.015 and 0.046, respectively; Fig. 1A, B, D and E), while *IL1B* and *TGFBRII* did not show significant differences (*p* > 0.050; Fig. 1C and F). *TNFA* and *IL6* showed a significant decrease in expression levels after bacterium eradication (*p* = 0.008 and *p* = 0.050, respectively; Fig. 1A and B), whereas *TGFBRII* expression was significantly increased (*p* = 0.029; Fig. 1F). Expression levels of *IL1B*, *IL12A* and *IL2* did not show significant differences before and after *H. pylori* eradication treatment (*p* = 0.130, 0.384 and 0.107, respectively; Fig. 1C–E) (Table S1 Supporting Information).

In addition, upon *H. pylori* eradication, we identified that *TNFA*, *IL1B* and *TGFBRII* mRNA expression levels were statistically similar to those found in the non‐infected patient group (Fig. 1A, C and F). However, *IL6*, *IL12A* and *IL2* mRNA expression levels remained significantly increased compared to the Hp− group (*p* = 0.001, 0.010 and 0.010, respectively; Fig. 1B, D and E). The presence of *cagA* genotype was not associated with gene expression in Hp+ patient samples (*p* > 0.05; Table S2 Supporting Information) and the *IL6* mRNA expression was the only statistically influenced by scoring of inflammation, which showed higher expression in the severe cases (*p* = 0.003).

### Protein expression: location, cell type and quantitative analysis

**Figure 2:**
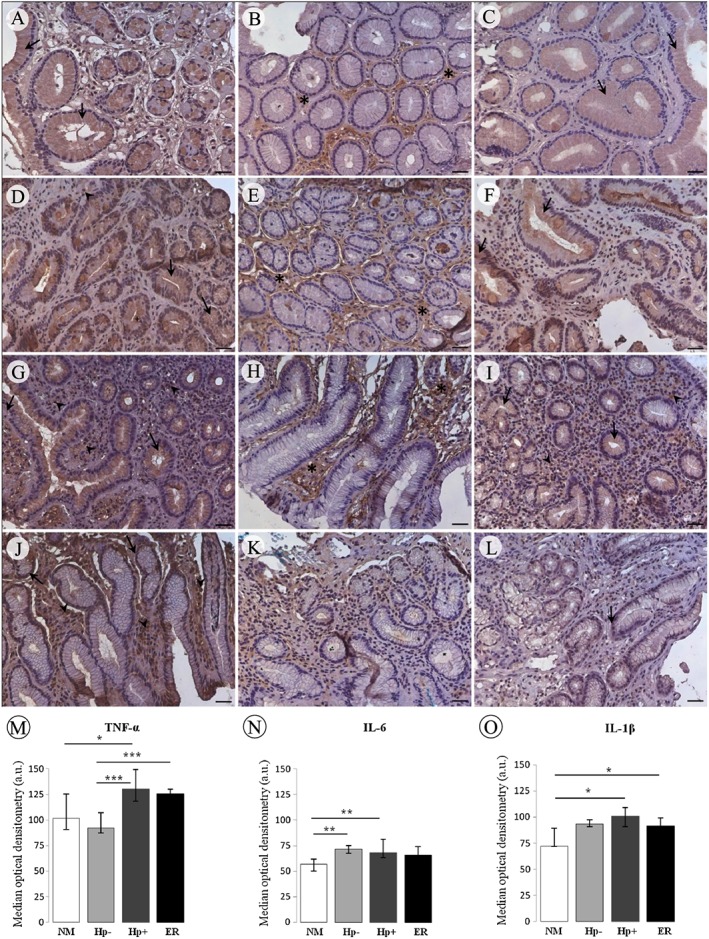
Immunoexpression of the proteins TNF‐α, IL‐6 and IL‐1β, considering negative (absent and weak) and positive (moderate and strong): normal gastric mucosa (A, B, C, respectively), chronic gastritis not infected with *H. pylori* (D, E, F, respectively) and chronic gastritis Hp+ before (G, H, I, respectively) and after eradication treatment (J, K, L respectively). In normal mucosa, TNF‐α (A) and IL‐1β (C) presented moderate to strong immunostaining in the cytoplasm of foveolar epithelium (arrow), while IL‐6 (B) showed strong immunostaining in the stroma (asterisk). In the Hp− group, IL‐1β (F) presented only immunostaining in the cytoplasm of foveolar epithelium (arrow), while TNF‐α (D) also showed weak immunostaining in inflammatory cells (arrowhead). For IL‐6, Hp− (E) and Hp+ before treatment groups (H) presented strong immunostaining in the stroma (asterisk). Before treatment, strong immunostaining was observed in both the foveolar epithelium (arrow) and in inflammatory cells (arrowhead) to TNF‐α (G) and IL‐1β (I). After eradication a similar immunostaining pattern those before treatment was found to TNF‐α protein (J), while IL‐1β, weak immunostaining was observed in foveolar epithelium (arrow). There was not stromal immunostaining of IL‐6 in most cases of eradicated group (K). Counterstain: Haematoxylin. Bars: 50 µm. Quantitative image analysis (M‐O) showed the cytoplasm immunostaining intensity (median ± interquartile range). a.u. arbitrary unit; * *p* ≤ 0.05; ** *p* ≤ 0.01; *** *p* ≤ 0.001.

**Figure 3:**
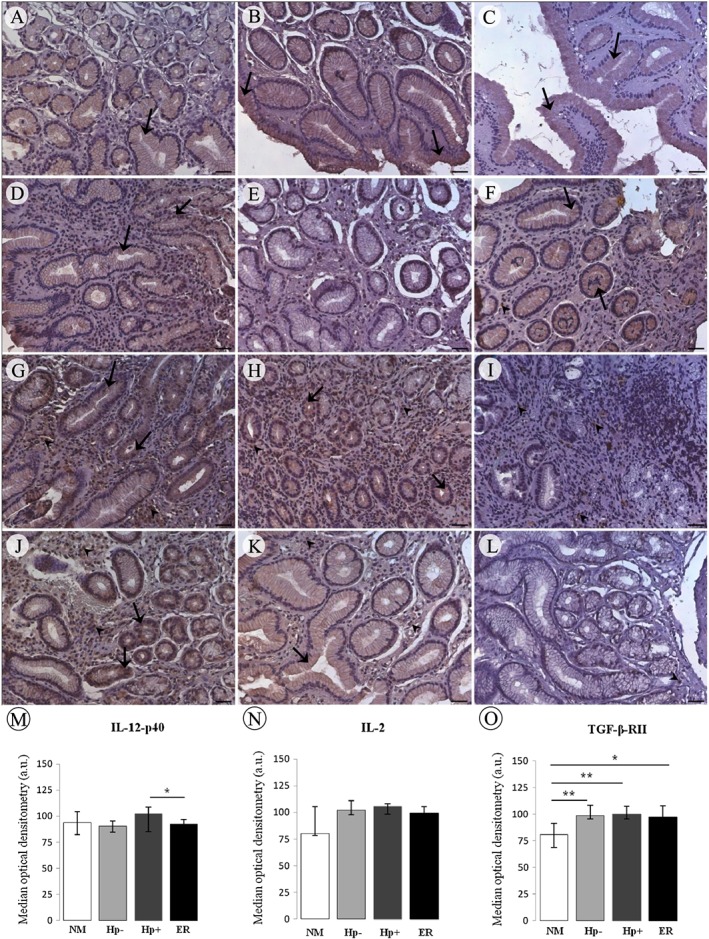
Immunoexpression of the proteins IL‐12p40, IL‐2 and TGF‐β‐RII, considering negative (absent and weak) and positive (moderate and strong): normal gastric mucosa (A, B, C, respectively), chronic gastritis not infected with *H. pylori* (D, E, F, respectively) and chronic gastritis Hp+ before (G, H, I, respectively) and after eradication treatment (J, K, L, respectively). In normal mucosa, IL‐12p40 (A), IL‐2 (B) and TGF‐β‐RII (C) presented mainly weak immunostaining in the cytoplasm of foveolar epithelium (arrow). The Hp− group presented predominance of weak or absent immunostaining in the epithelium (arrow) to IL‐12p40 (D) and IL‐2 (E) and strong to TGF‐β‐RII (F). For IL‐12p40 and IL‐2, Hp+ group (G and H, respectively) showed strong immunostaining in the cytoplasm of foveolar epithelium (arrow) and inflammatory cells (arrowhead) with a reduction in the IL‐2 expression in epithelium (K) and reduction in immunostaining intensity for IL‐12‐p40 after eradication (J). For TGF‐β‐RII, Hp+ group (I) presented weak immunostaining in the cytoplasm of foveolar epithelium and strong in inflammatory cells (arrowhead) with reduction after eradication only in the inflammatory cells (arrowhead) (L). Counterstain: Haematoxylin. Bars: 50 µm. Quantitative image analysis (M‐O) showed the cytoplasm immunostaining intensity (median ± interquartile range). a.u. arbitrary unit; * *p* ≤ 0.05; ** *p* ≤ 0.01; *** *p* ≤ 0.001.

Immunohistochemical analysis allowed us to show expression and cell localization of TNF‐α, IL‐1β, IL‐2, IL‐12‐p40 and TGF‐β‐RII proteins in the normal mucosa, and in *H. pylori* infected and non‐infected chronic gastritis. Expression of TNF‐α, IL‐1β, IL‐12‐p40, IL‐2 and TGF‐β type II receptor proteins was restricted to the cytoplasm of foveolar epithelium cells in non‐infected normal mucosa (Fig. 2A, C and 3A–C). Hp− chronic gastritis samples showed weak immunostaining in the foveolar epithelium for most cases of all proteins (Fig. 2D, F and 3D, E), except TGF‐β‐RII, which showed strong expression (Fig. 3F), besides immunostaining of inflammatory cells in some cases for TNF‐α and TGF‐β‐RII (Fig. 2D and 3F). However, in the Hp+ chronic gastritis group before treatment, most cases showed positive immunostaining for these proteins in both the cytoplasm of foveolar epithelium and in inflammatory cells (Fig. 2G, I and 3G–I). TNF‐α, IL‐2 and TGF‐β‐RII protein expression were significantly increased in inflammatory cells of the Hp+ group compared to Hp− group (*p* = 0.027, 0.013 and 0.004 respectively). TGF‐β‐RII positive immunostaining in the epithelium cells of Hp− group was more frequent than in Hp+ group (*p* = 0.009). The quantitative analysis did not show this difference for IL‐2 protein expression (Fig. 3N), although confirm significant difference for TNF‐α (*p* < 0.001) (Fig. 2M). For TGF‐β‐RII, greater frequency of positive and negative immunostaining cases in epithelium and inflammatory cells, respectively, in Hp− group in relation to Hp+ results in lack of difference in quantitative analysis (Fig. 3O).

IL‐6 protein showed a different immunostaining pattern, predominantly positive in the stroma in Hp+ and Hp− sample groups and in the normal mucosa (Fig. 2B, E, H), except in the ‘after eradication’ group, which showed predominance of negative cases (Fig. 2K). The quantitative analysis showed weaker levels of immunostaining of this protein compared to other and there was a predominance of expression in the stroma. This analysis showed that Hp− and Hp+ groups presented cytoplasm immunostaining more intense than normal mucosa (*p* = 0.008 and 0.007, respectively) (Fig. 2N).

Upon *H. pylori* eradication, expression of TNF‐α protein showed a similar pattern to Hp+ samples before treatment (Fig. 2J), whereas for IL‐1β and IL‐2 there was a reduction in the percentage of cases scored positive for protein expression in the cytoplasm of foveolar epithelium (Fig. 2L and 3K) and for TGF‐β‐RII and IL‐6 this reduction occurred in the inflammatory cells and stroma, respectively (Fig. 3L and 2K). These differences in specific regions of mucosa were not observed in the global quantitative analysis, although reduction in immunostaining intensity was obtained for IL‐12‐p40 (*p* = 0.013) (Fig. 3M). Immunostaining of IL‐2 and TGF‐β‐RII proteins differed significantly when we compared non‐infected and after eradication sample groups, wherein IL‐2 expression was significantly higher in inflammatory cells in the eradicated group (*p* = 0.035) and TGF‐β‐RII expression was increased in cytoplasm of epithelium cells in the Hp− group (*p* < 0.001). Quantitative analysis showed significant difference between these groups for TNF‐α immunostaining that was more intense in the eradicated group (*p* < 0.001) (Fig. 2M). Severe inflammation was related to more strong immunostaining of IL‐1β (*p* = 0.035) and IL‐2 (*p* = 0.026) proteins.

### Expression of miRNAs: miR‐103, miR‐181c, miR‐370 and miR‐375 is increased after bacterium eradication

**Figure 4:**
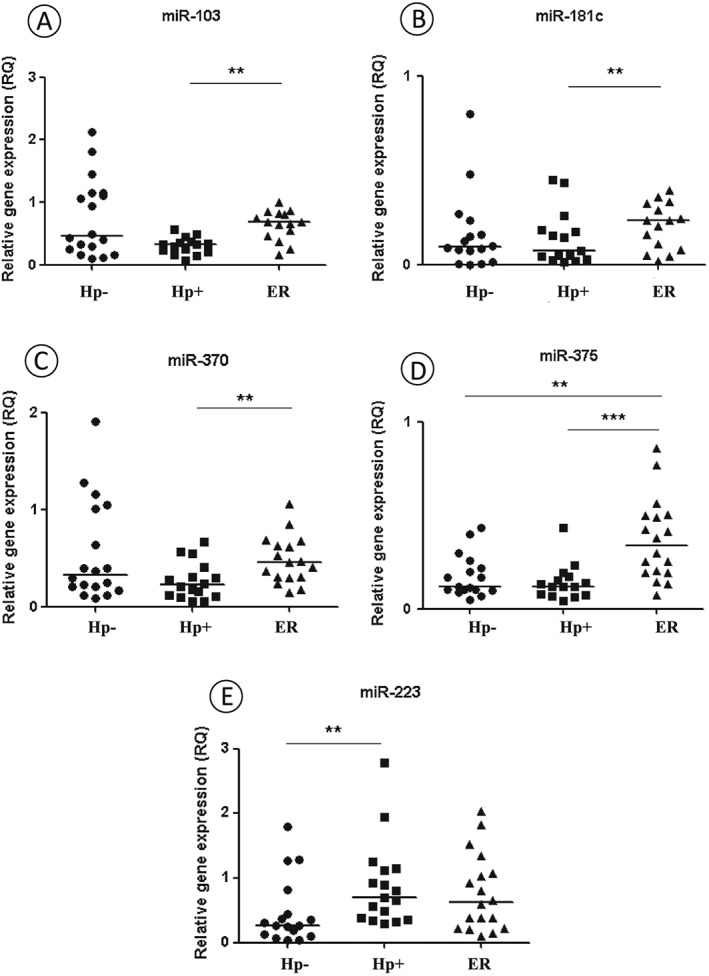
Relative expression of miRNAs: miR‐103 (A), miR‐181c (B), miR‐370 (C), miR‐375 (D) and miR‐223 (E) in patients with chronic gastritis *H. pylori‐*negative (Hp−) and *H. pylori‐*positive before (Hp+), and after eradication treatment (ER) groups. Data are presented as RQ values and each point represent one individual. Line symbolizes RQ median for each group. * *p* ≤ 0.05; ** *p* ≤ 0.01; *** *p* ≤ 0.001.

miRNA expression was also evaluated by quantitative real‐time PCR. Overall, miRNAs were down‐regulated in both Hp− and Hp+ groups without statistical difference. However miR‐103, miR‐181c, miR‐370 and miR‐375 showed a statistically significant increase in expression after bacterium eradication treatment (*p* = 0.003, 0.009, 0.018 and 0.001, respectively) (Fig. 4A–D). Except miR‐223, which showed a significantly higher expression in the Hp+ group before treatment compared to Hp− group (*p* = 0.009) (Fig. 4E). There was no significant difference between miR‐223 expression in patient samples before and after *H. pylori* eradication (*p* = 0.670) (Table S3 Supporting Information). In addition, miRNA expression levels were not associated with the *cagA* genotype in the Hp+ sample group before treatment (*p* > 0.05; Table S4 Supporting Information), although lower miR‐103 expression was observed in the severe inflammation degree in relation to mild and moderate score (*p* = 0.016).

### Principal Components Analysis (PCA)

In order to get an overview of the expression data for mRNA and miRNA and identify relevant genes between groups, we applied PCA analysis on covariance matrix. Overall median values of gene and miRNA expression in the Hp+ ‘before treatment’ group were higher than median values from Hp− samples (*p* = 0.001, Kruskal–Wallis Test) considering the first principal component. In addition, PCA showed that *IL6* was associated with the main differences between Hp+ and Hp− groups, representing 98% of the composition of the first principal component, because of its higher expression in Hp+ patients before treatment. Additionally, we also were able to detect a significant difference between Hp− and ‘after eradication’ treatment sample groups (*p* = 0.012), with higher overall median values of gene and miRNA expression in samples from ‘after eradication’ group. *IL6* expression was the largest contributor (44%) to this difference; followed by *IL12A*, *IL2* and *IL1B*. Expression of miRNAs and *TNFA* contributed very little to the difference between Hp− and eradicated sample groups.

### miRNA–mRNA interaction networks

Expression of inflammatory mediators may be regulated by specific miRNAs during the process of inflammation driven by *H. pylori* infection. Therefore, we investigated mRNA and miRNA expression levels in all sample groups, before and after treatment, and compared to non‐infected samples. Additionally, we identified miRNA–mRNA regulated networks that may be deregulated in gastritis and disease pathogenesis. In the Hp+ chronic gastritis before treatment group, *IL6* mRNA expression was negatively correlated with the expression of miR‐103 (*r* = −0.82; *p* < 0.001) and miR‐370 (*r* = −0.52; *p* = 0.048). *IL12A* mRNA expression was negatively correlated with miR‐103 (*r* = −0.60; *p* = 0.017), miR‐370 (*r* = −0.67; *p* = 0.006), miR‐181c (*r* = −0.53; *p* = 0.045) and miR‐375 (*r* = −0.51; *p* = 0.050) while *IL2* mRNA expression was negatively correlated with miR‐223 expression (*r* = −0.64; *p* = 0.010). In the non‐infected chronic gastritis group, miR‐223 was also negatively correlated with *IL1B* mRNA expression (*r* = −0.52; *p* = 0.048). Interestingly, upon bacterium eradication treatment, the correlation between *IL6* and miR‐103 was now positive (*r* = 0.62; *p* = 0.019).

Further, we have analysed the correlation between expression of mRNA and miRNA before and after bacterium eradication treatment; this analysis showed that *TNFA* expression is inversely associated with miR‐103 (*p* = 0.002), miR‐181c (*p* = 0.004), miR‐223 (*p* = 0.026), miR‐370 (*p* = 0.002) and miR‐375 (*p* = 0.001). This finding indicates that while mRNA expression decreases after bacterium eradication treatment, expression of several miRNA increases.

**Figure 5:**
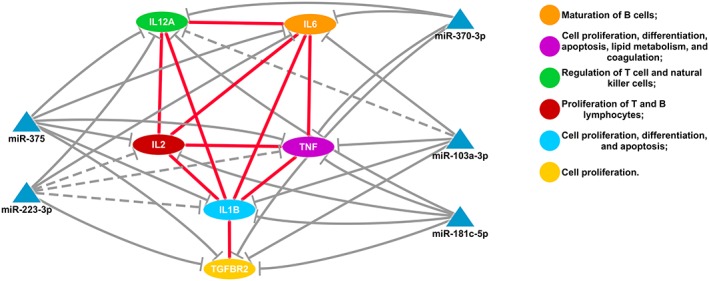
Protein interaction network showing miRNAs and their predicted gene targets. The protein interaction network (red lines) shows the interaction between proteins encoded by target genes that are predicted to be regulated by miRNAs. Predicted interactions between miRNAs and target genes are shown by the continuous gray lines. Dotted gray line represents some interaction possibilities obtained by experimental validation in this study between, for example, miR‐103 and the gene *IL12A* and between mir‐223 and genes *IL2*, *TNFA* and *IL1B.* Ellipses represent target genes/proteins; blue triangles represent miRNAs.

*In silico*, miRNA–mRNA interaction network analysis showed relationships between genes encoding inflammatory mediators and between genes and miRNAs (Fig. 5). Negative correlations between the expression of mRNA and miRNA found in this study are demonstrated in this network (Fig. 5—continuous gray line), although some interactions were not validated in our study. Furthermore, our results indicated other possibilities of interaction (Fig. 5—dotted gray line), such as miRNAs miR‐103 interacting with *IL12A* and miR‐223 interacting with *IL2*, *TNFA* and *IL1B.*

## Discussion

To the best of our knowledge, ours is the first study to investigate the effect of eradication treatment of *H. pylori* on mRNA and protein expression levels of inflammatory mediators (*TNFA*, *IL6*, *IL1B*, *IL12A*, *IL2* and *TGFBRII*) as well as the interaction with miRNAs involved in regulation of inflammation and tumorigenesis processes, such as miR‐103, miR‐181c, miR‐370, miR‐375 and miR‐223 in patients with chronic gastritis and infected by *H. pylori*. We observed that *H. pylori* infection, regardless of *cagA* genotype, increases mainly the pro‐inflammatory cytokines *TNFA* and *IL6* expression in gastric mucosa, especially in the inflammatory cells and stroma, and its eradication partially reduces the inflammatory response. However, gastric mucosa upon bacterium eradication does not show the same transcriptional pattern as in the absence of *H. pylori* infection. Furthermore, the relationships between the expression of mRNAs and miRNAs suggest that expression of cytokines is modulated by miRNAs especially in the presence of *H. pylori* and its eradication increases miRNA expression, which may influence the reduction of inflammatory response. miRNAs may thus participate in the pathological process triggered by *H. pylori* in the gastric mucosa, influencing host inflammatory response against infection.

*H. pylori* infection results in increased expression of *TNFA*, *IL6*, *IL12A* and *IL2* in the gastric mucosa, in addition to the effect of chronic gastritis, and for TNF‐α and IL‐2 proteins, this increase was mainly observed in inflammatory cells. Our data showed that 3 months after bacterium eradication treatment, there was a decreased expression of *TNFA* and *IL6* mRNA and IL‐12‐p40 protein and increased expression of *TGFBRII* mRNA, in addition to the significant reduction in the inflammation degree, showing that the inflammatory response is reduced, once TNF‐α and IL‐6 cytokines are related to induction of inflammation (Szlosarek and Balkwill, 2003; Rincon, 2012), IL‐12 binds to innate immunity with adaptive by favouring Th_1_ response (Del Vechio *et al*., 2007) and TGF‐β‐RII is one of the main receptors of anti‐inflammatory cytokine TGF‐β (Achyut and Yang, 2011). However, this reduction in inflammatory response was not complete, because expression of *IL1B* and *IL2* was not significantly changed after bacterium elimination. Although IL‐1β is related to innate immune response (Lopez‐Castejon and Brough, 2011), IL‐2 acts on T cells (Liao *et al*., 2011), so that *H*. *pylori* eradication failure to restore the expression of this cytokines related to adaptive response. Mera *et al*. (2005) showed that chronic inflammation continued present, but significantly lower, 12 years after *H. pylori* eradication treatment. In addition, as well as our results, studies have shown a significant reduction in infiltration of neutrophils and mononuclear cells (Lee *et al*., 2010), but permanence of *H. pylori*‐specific Th_17_ cells after treatment (Serelli‐Lee *et al*., 2012) evidencing the maintenance of the adaptive immune response cells. Taken together, these data indicate that inflammatory response does not disappear completely after pathogen eradication.

The TGF‐β/TGF‐β‐RII signalling pathway has been show to comprise a tumour suppressor pathway (Hahm *et al*., 2002; Takeno *et al*., 2002) deregulated in gastric cancer (Li *et al*., 2015). Dominant negative mutant TGF‐β‐RII mice infected by *H. pylori* has been shown to present greater cell proliferation, and to develop gastric adenocarcinoma in the same time frame that wild type mice develop chronic gastritis only. This finding indicates that normal TGF‐β signalling can suppress or delay gastric carcinogenesis (Hahm *et al*., 2002). Moreover, reduction in the TGF‐β‐RII expression can lead to deregulation in the signalling pathway impairing homeostasis and increasing tissue inflammation (Hong *et al*., 2010). Therefore, an increase in *TGFBRII* mRNA expression after *H. pylori* eradication, as observed in our study, may indicate that eradicated patients respond more efficiently to anti‐inflammatory cytokine TGF‐β, having less severe inflammation and better prognosis than Hp+ patients.

The intensity of the remaining inflammatory response may also depend on the time that the individual remained infected before treatment (Romero‐Gallo *et al*., 2008). In this respect, most individuals in developing countries are infected during childhood (Kodaira *et al*., 2002; Miranda *et al*., 2010), thus is plausible that patients evaluated in our study are longtime chronic infection carriers, which may have hindered the response to treatment for restoring cytokine expression evaluated. Furthermore, our data show that mRNA expression of *IL6*, *IL12A* and *IL2* in the eradicated group was higher than in the group of patients without history of *H. pylori* infection, while TGF‐β‐RII protein expression in epithelium cells was higher in this non‐infected group. This finding suggests that gastric mucosa after bacterium elimination does not harbour the same transcriptional patterns as in absence of infection, even both presenting chronic gastritis, suggesting that changes in the expression of some cytokines, caused by *H. pylori* infection, are maintained in the gastric mucosa even after its eradication.

Cytokines and their receptors are not the only mediators involved in chronic inflammation process triggered by *H. pylori* infection. Recent studies have shown that the pathogen affects miRNA global expression profiles in the gastric mucosa, related to the host immune and inflammatory responses (Noto and Peek, 2012; Link *et al*., 2015). Therefore, miRNAs miR‐103, miR‐181c, miR‐370, miR‐375 and miR‐223 were examined herein because of their involvement in the regulation of inflammatory processes and gastric carcinogenesis.

Overall, our results showed that miRNAs miR‐103, miR‐181c, miR‐370 and miR‐375 are down‐regulated in chronic gastritis regardless of *H. pylori* infection, because both infected and non‐infected sample groups had reduced expression of these miRNAs. Other studies have shown down‐regulation of miRNAs in inflammatory gastric lesions infected or non‐infected by *H. pylori* (Matsushima *et al*., 2011; Shiotani *et al*., 2012); however, in gastric cancer some studies have reported increased expression of miRNAs such as miR‐103, miR‐181c, miR‐370, miR‐21 and miR‐146a (Wu *et al*., 2011; An *et al*., 2013; Fan *et al*., 2013; Ishimoto *et al*., 2016). This different expression profile between gastric cancer and *H. pylori* infected gastric mucosa suggests that these miRNAs might act differently in the distinct stages of gastric tumorigenesis and disease progression.

Matsushima *et al*. (2011) showed that 65% of miRNAs evaluated in *H. pylori* infected noncancerous gastric mucosa were down‐regulated compared to gastric mucosa from non‐infected individuals. In addition, expression of some miRNAs was inversely correlated with the degree of mononuclear cells infiltration indicating chronic inflammation. Similarly we also observed lower expression of miR‐103 associated with severe inflammation degree in Hp+ group before treatment. This way chronic gastritis regardless of *H. pylori* infection can have a negative effect on the expression of these miRNAs, but the infection by *H. pylori* should have an additional effect on miRNA expression levels, once significant increases in the relative expression were observed after bacterium eradication. Meanwhile, previous reports indicate that bacterium eradication improved in the expression levels of miRNAs only in early lesion of gastric carcinogenesis cascade, but did not in intestinal metaplasia and gastric cancer (Shiotani *et al*., 2012).

Unlike the other evaluated miRNAs, miR‐223, which is considered an oncogene associated with formation of gastric tumours (Li *et al*., 2012), showed higher expression in the Hp+ group before treatment compared to the non‐infected group, but no significant difference was observed after eradication. miR‐223 was closely associated with neutrophil infiltration in the *H. pylori* infected gastric mucosa (Matsushima *et al*., 2011), although in the present study its expression was not related to the inflammation degree. A recent review by Ishimoto *et al*. (2016) confirms the involvement of miRNA changes identified herein, such as downregulation of miR‐375 and miR‐181c in gastric cancer tissue associated with cell growth, downregulation of miR‐370 involved in *H. pylori*‐induced progress of gastritis to gastric cancer, miR‐223 overexpression associated with stimulation and migration of non‐metastatic gastric cancer cells and the roles of miRNAs miR‐223, miR‐375 and miR‐103 as potential biomarkers in this neoplasm. Therefore, *H. pylori* and its eradication treatment may change miRNA expression profiles and consequently miRNAs may influence the pathogenesis of this bacterium in gastric mucosa.

Deregulated miRNA expression caused by *H. pylori* may contribute to destabilize host immune response (Noto and Peek, 2012); one possible mechanism is through the action of these miRNAs on target mRNAs encoding pro‐ and anti‐inflammatory cytokines (Xu *et al*., 2011). When we assessed the relationships between expression of mRNAs and miRNAs, we observed that most of the negative correlations occur in the Hp+ group before treatment. Expression of *IL6* mRNA was negatively correlated with miR‐103 and miR‐370. In addition, *IL12A* mRNA expression showed negative correlation with miR‐103, miR‐181c, miR‐370 and miR‐375, while the expression of *IL2* mRNA was correlated with miR‐223 expression also in the Hp+ group before treatment. On the other hand, in the non‐infected group only correlation between miR‐223 and *IL1B* mRNA was observed. Our results suggest that cytokine expression may be modulated by miRNAs especially in the *H. pylori* presence, so that bacterium infection can alter miRNA expression in gastric mucosa and this leads to changes in the expression of such inflammatory mediators, which consequently influences the host response against infection. Isomoto *et al*. (2012) also found differences in the connection between expression of cytokines and miRNAs in relation to *H. pylori* status. Furthermore, *H. pylori* was able to promote strong inflammation and miRNA expression response in a co‐culture cell system and in this context increased TNF‐α expression was related to function loss of miR‐155 (Hocès de la Guardia *et al*., 2013). Notably, in our study the correlation between expression of *IL6* mRNA and miR‐103 was negative in *H. pylori* infected patients and positive in eradicated patient samples. This strengthens the idea that these miRNAs and inflammatory mediators are likely associated with the pathological process in the presence of *H. pylori*, but not influence in the infection absence. The analysis of miRNA–mRNA interaction networks also reinforces these findings, evidencing that proteins encoded by deregulated genes interact with each other and with the miRNAs in the inflammatory cascade triggered by *H. pylori.* Furthermore, interaction networks identified in our study demonstrated possible relationships that may be further assessed by functional analysis.

Our results did not evidence influence of *cagA* genotype on mRNA and miRNA expression nor on bacterial eradication rate. Some studies have indicated that the secretion of cytokines IL‐8, TNF‐α and IL‐1β and the mRNA expression of IL‐12 are higher in individuals infected with *cagA*+ strains (Yamaoka *et al*., 1997; Hida *et al*., 1999; Kumar Pachathundikandi *et al*., 2011; Mustapha *et al*., 2014). On the other hand, other studies showed that the presence of this genotype did not influence the expression of *TNFA* (Zalewska‐Ziob *et al*., 2009) and *IL12A* (Bauditz *et al*., 1999) nor of proteins IL‐6, IL‐12, TNF‐α and IL‐1β (Kranzer *et al*., 2005; García‐González *et al*., 2009). In relation to miRNAs, an association between *cagA* genotype and reduction in miR‐370 expression has been reported (Feng *et al*., 2013), which was not found in herein. In this context, we must consider some limitations of our study, because of the reduction of the number of patients in the subgroup stratification, which may have contributed during the statistical analysis for this parameter.

The different analyses performed in our study, in addition to bioinformatics analysis of miRNA–mRNA networks has highlighted *IL*6 and *TNFA* which contributed to major differences in mRNA expression between infected and non‐infected groups, and also after bacterium eradication. Regarding miRNAs, miR‐103, miR‐181c, miR‐370 and miR‐375 stood out in the context of *H. pylori* eradication, while miR‐223 was influenced by the presence of this pathogen.

IL‐6 is the most abundant cytokine during prolonged and uncontrolled activation of inflammation (Mauer *et al*., 2015). Overexpression of IL‐6 was reported in *H. pylori* infection by others studies (Zhao *et al*., 2010; Zhang *et al*., 2014). Concerning treatment effect, Ando *et al*. (1998) showed that IL‐6 expression assumes values close to Hp− individuals six months after bacterial eradication in patients with duodenal ulcer, which may indicate that a longer time than used in this study would result in a restoration of this cytokine expression. However, other study showed that IL‐6 concentration was not elevated in individuals who had bacterium infection in the past, so that this cytokine would not influence the maintenance of *H. pylori*‐specific Th_17_ cells in the gastric mucosa after eradication (Serelli‐Lee *et al*., 2012). Our study showed that IL6 expression, although reduced in comparison with Hp+ individuals, was higher in the eradicated group that in non‐infected group suggesting that this interleukin may be cooperating with the differentiation and permanence of Th_17_ cells after bacterium elimination. Differentiation of Th_17_ cells of IL‐6‐dependent manner was demonstrated in gastric myofibroblasts/fibroblasts isolated from infected gastric cancer mucosa (Pinchuk *et al*., 2013).

TNF‐α plays an important role in mediating chronic inflammation, and it is a key tumour promoter (Szlosarek and Balkwill, 2003). Overexpression of *TNFA* was observed in *H. pylori* infected patients compared with non‐infected peptic ulcer (Goll *et al*., 2007), as well as during progression of gastric cancer (Zhao *et al*., 2010; Senthilkumar *et al*., 2011). After eradication, reduction in *TNFA* expression was related to reduction in infiltration of neutrophils and mononuclear cells (Lee *et al*., 2010). Furthermore, *H. pylori* presents a virulence factor named Tip‐α (*TNF‐α‐inducing protein*) able to strongly induce the TNF‐α expression by NF‐κB activation, so that elimination of this bacterium results in induced weak expression of this cytokine in the host (Suganuma *et al*., 2012). In addition, to reinforce the regulatory role of miRNAs on cytokine expression in the presence of *H. pylori* infection, our study evidenced that *TNFA* expression decreased after bacterium eradication associated with increased expression of miR‐103, miR‐181c, miR‐223, miR‐370 and miR‐375. This finding suggests that *H. pylori* eradication can lead to an increase in miRNA expression, and this would lead to reduction of inflammatory response.

Accordingly, our results evidence that *H. pylori* and its eradication interfere on the expression levels of certain cytokines and miRNAs that regulate the inflammatory process in early gastric lesions and also reverse more severe lesions as atrophic gastritis associated or not with the metaplasia for less serious lesions and even normal mucosa, as observed in 24% of our samples, as well as the significant reduction in the inflammation degree after eradication Furthermore, our findings suggest that miRNAs may be an intermediary in the molecular mechanism leading to inflammation in gastric lesions in response to *H. pylori* infection, so that functional studies are required to confirm their role in such pathways. A better understanding of the molecular mechanisms of regulation of host immune and inflammatory response against *H. pylori* will help in the development of vaccines or more effective therapeutic strategies.

### **Experimental procedures**

This study was approved by the local Research Ethics Committee (CEP‐IBILCE/UNESP, number 307.691/2013), and written informed consent was obtained from all individuals.

### Clinical Samples

Gastric biopsies from the antrum region were collected during upper endoscopy in the Ambulatory of Gastro‐Hepatology at the Base Hospital and João Paulo II Hospital, both in São José do Rio Preto, SP, Brazil, between May 2010 and December 2012. All specimens were kept in RNA*later*® solution (*Applied Biosystems*) and stored at −20 °C until nucleic acid extraction. Histological analyses for *H. pylori* diagnosis and histopathological classification of lesions were done according the Sydney system (Dixon *et al*., 1996). Patients with gastric cancer, infectious diseases or inflammatory processes and who had taken any antibiotics, nonsteroidal anti‐inflammatory drugs, or corticosteroids two months prior to endoscopy, and/or proton‐pump inhibitors, H_2_ antagonists 10–15 days prior to endoscopy were excluded from the study. A total of 55 patients were included in the study, 31 diagnosed with gastric dyspepsia *H. pylori‐*positive (Hp+), 20 with gastric dyspepsia *H. pylori‐*negative (Hp−) without previous infection registration and four with histologically normal gastric mucosa and *H. pylori‐*negative (Hp−) which were used as controls for gene and miRNA expression (RT‐qPCR) and immunohistochemical (IHC) analyses. Patient's information including demographic characteristics was obtained after patients filled out a standard questionnaire containing questions about smoking and drinking habits, previous or ongoing treatment, medication use, previous surgeries and family history of gastrointestinal diseases. In all patient groups, the majority was of female gender, had no drinking and no smoking habits (Table 1). Regarding histopathological classification, most patients had chronic gastritis only, and the inflammation scoring was evaluated in all cases for expert (K.R.M.L.) based on the inflammatory infiltrate in three degrees: mild, moderate and severe. In the Hp− group, 10% of patients had metaplastic chronic gastritis, while the Hp+ before treatment group had atrophic gastritis and metaplastic atrophic gastritis in 23% and 16% of individuals respectively.

**Table 1:** Epidemiological data of individuals with normal gastric mucosa without *H. pylori* infection (Hp−), *H. pylori‐*negative (Hp−) chronic gastritis patients and *H. pylori‐*positive (Hp+) chronic gastritis patients before treatment.

Variable	Normal mucosa (Hp−) N (%)	Hp− N (%)	Hp+ N (%)
**Gender**			
Female	3 (75)	14 (70)	19 (61.3)
Male	1 (25)	6 (30)	12 (38.7)
**Total**	4	20	31
			
**Age (years) Mean ± standard deviation**	30 ± 12.50	52 ± 19.24	44 ± 12.00
	**<30** 3 (75)	**<52** 6 (33,3)	**<44** 12 (46,2)
	**≥30** 1 (25)	**≥52** 12 (66,7)	**≥44** 14 (53,8)
**Total**	4	18*	26*
			
**Smoking**			
Yes	0 (0)	4 (23.5)	10 (33.3)
No	4 (100)	13 (76.5)	20 (66.7)
**Total**	4	17*	30*
			
**Drinking**			
Yes	0 (0)	6 (35.3)	9 (30)
No	4 (100)	11 (64.7)	21 (70)
Total	4	17*	30*
			
**Histology**			
Chronic gastritis		18 (90)	19 (61.3)
Atrophic gastritis		0 (0)	7 (22.6)
Metaplastic atrophic/chronicgastritis		2 (10)	5 (16.1)
**Total**		20	31

* Parameter not available for some individuals; N = sample number.

*H. pylori‐*positive patients were subjected to standard triple therapy for bacterium eradication consisting of amoxicillin (1 g), clarithromycin (500 mg) and omeprazole (20 mg) all twice daily for seven days. About three months after treatment, patients underwent a second endoscopy exam to evaluate bacterium eradication status. Among 31 Hp+ treated patients, 21 of them eradicated the bacterium (68%) and 10 remained infected (32%) after treatment. After eradication, a reversal of histophatological lesions for less aggressive types, including restoration of normal mucosa, was observed in 24% of cases.

### Nucleic acid extraction, molecular diagnosis of H. pylori *and of* cagA *virulence genotype*

DNA and RNA of gastric biopsies were extracted according to the TRIzol® reagent (*Invitrogen*) protocol, and quantifications were performed using NanoDrop® ND‐1000 Spectrophotometer (*Thermo Scientific*). RNA integrity was assessed by the presence of 18S and 28S ribosomal RNA (rRNA) subunits in 1.0% agarose gel electrophoresis.

DNA samples were subjected to multiplex PCR as previously described by us (Rossi *et al*., 2014) to confirm the histopathological diagnosis of *H. pylori* infection. Samples with discordant results between molecular and histopathological diagnosis were excluded from the study. *H. pylori‐*positive samples were subjected to a second PCR run, to investigate the presence of *cagA* virulence genotype. We used primers for the *cagA* gene (Rossi *et al*., 2014) and for the 16S rRNA with specificity for *H. pylori* (Scholte *et al*., 1997) to confirm the efficiency of the PCR reaction. Reactions contained 1X Buffer, 1.2 mM of each deoxyribonucleotide, 1.0 mM MgCl_2,_ 0.4 mM of each primer (*Invitrogen*), 1.5U Platinum *Taq* DNA Polimerase (*Invitrogen*) and 300 ng of DNA, in a 25 µl final volume. Reaction conditions were: 94 °C for 5 min for DNA denaturation, 40 cycles of 1 min at 94 °C, 1 min at 56 °C and 1 min at 72 °C and, for final extension, 7 min at 72 °C.

### mRNA and microRNA expression by quantitative real‐time PCR (RT‐qPCR)

Reverse Transcription (RT) reaction for genes was performed with High Capacity cDNA Archive Kit (*Applied Biosystems*) according to the manufacturer's protocol. The synthesis of complementary DNA (cDNA) to the miRNAs was carried out in multiplex mode with TaqMan® MicroRNA Reverse Transcription Kit (*Applied Biosystems*). Each reaction contained 1X RT Buffer, 2 mM dNTPs with DTTP, 150U MultiScribe Reverse Transcriptase, 3.8U RNase Inhibitor, 6 µl pool containing RT primers (5X) of all miRNAs available in the TaqMan® MicroRNA Assay (*Applied Biosystems*) and 300 ng of total RNA, in a 15 µl final volume. Thermocycler conditions were 16 °C for 30 min, 42 °C for 30 min and 85 °C for 5 min Before qPCR, synthesized cDNA for cytokines genes and miRNAs were subjected to specific multiplex pre‐amplification with TaqMan® PreAmp Master Mix (*Applied Biosystems*).

qPCR was performed in a *StepOnePlus Real Time PCR System 2.2.3* (*Applied Biosystems*), using specific TaqMan® probe for target genes *TNFA* (Hs01113624_g1), *IL1B* (Hs01555410_m1), *IL6* (Hs00985629_m1), *IL12A* (Hs00168405_m1), *IL2* (Hs00174114_m1) and *TGFBRII* (Hs00234253_m1) (*Applied Biosystems Inc*) and two reference genes, *ACTB* (*Catalog*#: *4352935E*) and *GAPDH* (*Catalog*#: *4352934E*), and for target miRNAs hsa‐miR‐103a‐3p (MIMAT0000101; Hs000439), hsa‐miR‐181c‐5p (MIMAT0000258; Hs000482), hsa‐miR223‐3p (MIMAT0000280; Hs002295), hsa‐miR‐370‐3p (MIMAT0000722; Hs002275) and hsa‐miR‐375 (MIMAT0000728; Hs000564) and for two miRNA endogenous controls; RNU6B (Hs001093) and RNU48 (Hs001006) (*Applied Biosystems Inc*). All reactions were performed in triplicate. Protocols followed the manufacturer's instructions (*Applied Biosystems* and *Promega* respectively). Relative quantification (RQ) of mRNA and miRNA expression were calculated using the 2^−ΔΔCt^ method according to the model proposed by Livak and Schmittgen (2001) and normalized to the reference control genes and a pool of Hp− normal mucosa samples which was used as a calibrator. The RQ was expressed the median expression value of each gene or miRNA relative to the control samples. Quantitative PCR experiments followed the MIQE guidelines (Bustin *et al*., 2009).

### Immunohistochemistry assay

Immunohistochemical analysis was performed on samples from normal gastric mucosa, Hp− chronic gastritis and samples from the Hp+ chronic gastritis before and after *H. pylori* eradication. Deparaffinized and rehydrated tissue slides were subjected to antigen retrieval in Sodium Citrate Buffer pH 6.0, using a high‐temperature antigen‐unmasking technique, and subsequent inactivation of endogenous peroxidase with hydrogen peroxide 3%. Initially, sections were incubated with specific primary antibodies at 4 °C overnight: rabbit polyclonal TNF alpha antibody (NB600‐587, 1:200 dilution; *Novus Biologicals*), rabbit polyclonal IL1 beta antibody (NB600‐633, 1:200 dilution; *Novus Biologicals*), rabbit polyclonal IL6 antibody (NB600‐1131, 1:400 dilution; *Novus Biologicals*), rabbit monoclonal IL2 antibody (NBP1‐40687, 1:250 dilution; *Novus Biologicals*), rabbit polyclonal TGF beta Receptor II antibody (NBP1‐19434, 1:100 dilution; *Novus Biologicals*) and rabbit monoclonal IL‐12 alpha antibody (TA310616, 1:500 dilution; *OriGene*). Slides were then incubated with biotinylated secondary antibody and HRP polymer conjugate of the commercial kit PicTure^TM^‐MAX Polymer (*Invitrogen*) for 30 min each. 3,3′Diaminobenzidine tetrahydrochloride (DAB) containing 0.005% H_2_O_2_ was used for immunostaining and haematoxylin as the counterstain. All experiments had a negative control consisting of a section incubated only with primary antibody diluent (*Leica*) without the primary antibody.

Slides were evaluated by an expert pathologist (K.R.M.L.). Epithelial cells cytoplasm and inflammatory cells were assessed for proteins TNF‐α, IL‐1β, IL‐12p40, IL‐2 and TGF‐β‐RII, whereas for IL‐6 were evaluated epithelial cells cytoplasm and tissue stroma. Protein expression was classified as negative when the staining was absent or weak and positive when moderate or strong. Figures were obtained with AxioVision software under a Zeiss‐Axioskop II light microscope (*Carl Zeiss*). Furthermore, immunostaining was quantitatively assessed, in double blind, using image analysis software Image J of National Institutes of Health (EUA) (http://imagej.nih.gov/ij/). Initially, figures were subjected to colour deconvolution trough plugin IHC Profiler (Varghese *et al*., 2014) and subsequently measured the mean gray value of the interest region in the DAB picture. Three figures in each case were assessed. The arbitrary scale varies from 0 to 255.

### miRNA–mRNA interaction networks

Prediction of targets regulated by miRNAs was performed using the bioinformatics tool microRNA Data Integration Portal (http://ophid.utoronto.ca/mirDIP/) (Shirdel *et al*., 2011). A protein–protein interaction network was generated via String (version 9.1) (Franceschini *et al*., 2013) and GeneMANIA (version 3.2) (Montojo *et al*., 2010) databases, using the target genes as input. miRNAs and target genes identified have been integrated into interaction networks, for this, Cytoscape software (version 3.1.1) (Chen *et al*., 2012) was applied for visualization and analysis of network. The biological function of genes was identified using the tool BiNGO in Cytoscape (version 3.0.2) (Maere *et al*., 2005). Network is illustrated as graphs with the nodes representing the genes/miRNAs/proteins and the edges representing their interactions.

### Statistical analysis

Data were analysed using the box‐plot graphic method for outliers detection (Williamson *et al*., 1989), which were removed from subsequent analyses. The distribution of continuous data was evaluated using the D'Agostino and Pearson normality test. Wilcoxon Signed Rank test was used to assess changes in mRNA or miRNA expression in relation to a pool of Hp− normal mucosa samples, while Mann–Whitney test was used for comparisons of mRNA, miRNA and quantitative protein expression between groups and to associate the influence of *cagA* genotype in mRNA and miRNA expression levels and of the scoring of the inflammation in mRNA, miRNA and quantitative protein expression. Fisher Exact Test was employed to evaluate the association between *cagA* genotype with eradication rate and to compare protein expression between the groups, comparing the number of positive and negative cases for each group, while Chi‐square test was used by compared the scoring of inflammation between groups. Wilcoxon matched pairs test and McNemar test were used to compare mRNA/miRNA/quantitative protein expression and qualitative protein expression between Hp+ before and after eradication treatment respectively. The correlation between mRNA and miRNA expression within each group was analysed using Spearman's correlation. Correlation between mRNA and miRNA expression in Hp+ before and after eradication treatment was analysed by calculating the differences between RQ values using one sample *t* test. Principal Component Analysis (PCA) was used to find the key mediators involved in the difference between the groups with subsequent Kruskal–Wallis test. Values *p* < 0.05 were considered significant.

## Conflict of interests

The authors declare no conflicts of interest.

## Supporting information


**Table S1.** Relative expression of *TNFA*, *IL6*, *IL1B*, *IL12A*, *IL2* and *TGFBRII* mRNA in *H. pylori*‐negative (Hp−) chronic gastritis patients and *H. pylori*‐positive (Hp+) chronic gastritis patients before and after eradication.
**Table S2.** Comparisons between the relative expression of evaluated genes according with *cagA* genotype in *H. pylori*‐positive (Hp+) chronic gastritis patients before treatment.
**Table S3.** Relative expression of miR‐103, miR‐181c, miR‐370, miR‐375 and miR‐223 in *H. pylori*‐negative (Hp−) chronic gastritis patients and *H. pylori*‐positive (Hp+) chronic gastritis patients before and after eradication.
**Table S4.** Comparisons between the relative expression of evaluated miRNAs according with *cagA* genotype in *H. pylori*‐positive (Hp+) chronic gastritis patients before treatment.

Supporting info item
