# Supplementary material for: Interaction between inflammatory mediators and miRNAs in Helicobacter pylori infection
Source: Cell Microbiol. 2016 Apr 14;18(10):1444–58. doi: 10.1111/cmi.12587 (PMC5074252; doi:10.1111/cmi.12587)
Supplement: Supplementary file 1 — Supporting info item [file CMI-18-1444-s001.zip › Supplemetary material.pdf]

Table S1: Relative expression of *TNFA, IL6, IL1B, IL12A, IL2* and *TGFBRII* mRNA in *H. pylori*-negative (Hp-) chronic gastritis patients and *H. pylori*-positive (Hp+) chronic gastritis patients before and after eradication.

Genes	Hp-	Before Treatment (Hp+)	After eradication (Hp-)
TNFA			
RQ median	1.35	2.58	1.44
Range	0.38-4.26	0.49-4.65	0.54-3.07
p	**0.024***		**0.008***
IL6			
RQ median	0.74	7.39	2.90
Range	0.15-4.81	0.37-32.02	0.63-12,90
p	**<0.001***		**0.050***
IL1B			
RQ median	1.58	1.78	2.40
Range	0.61-3.07	0.98-7.44	0.93-8.98
p	0.569		0.130
IL12A			
RQ median	0.87	2.00	1.98
Range	0.17-3.13	0.39-5.01	0.67-9.53
p	**0.015***		0.384
IL2			
RQ median	0.35	0.75	1.04
Range	0.05-2.49	0.23-1.84	0.21-11.56
p	**0.046***		0.107
TGFBRII			
RQ median	0.67	0.75	0.80
Range	0.28-1.82	0.32-1.50	0.32-2.90
p	0.964		**0.029***

RQ = Relative Quantification; p: Mann-Whitney test or Wilcoxon matched pairs test;
* significant difference

Table S2: Comparisons between the relative expression of evaluated genes according with *cagA* genotype in *H. pylori*-positive (Hp+) chronic gastritis patients before treatment.

Gene	*cagA*+		*cagA*-
TNFA			
RQ median	3.32		3.25
Range	1.91-4.49		2.25-4.65
p		0.833	
IL6			
RQ median	12.24		10.92
Range	3.05-21.59		4.22-32.02
p		1.000	
IL1B			
RQ median	1.44		2.84
Range	0.98-4.39		1.24-7.44
p		0.240	
IL12A			
RQ median	2.10		2.27
Range	1.48-2.76		1.36-5.01
p		0.628	
IL2			
RQ median	0.61		1.04
Range	0.28-1.02		0.30-1.84
p		0.310	
TGFBRII			
RQ median	0.61		0.76
Range	0.32-1.50		0.42-1.19
p		0.295	

RQ = Relative Quantification; p: Mann-Whitney test

Table S3: Relative expression of miR-103, miR-181c, miR-370, miR-375 and miR-223 in *H. pylori*-negative (Hp-) chronic gastritis patients and *H. pylori*-positive (Hp+) chronic gastritis patients before and after eradication.

microRNA	Hp-	Before Treatment (Hp+)	After eradication (Hp-)
**miR-103**			
RQ median	0.46	0.33	0.68
Range	0.10-2.13	0.07-0.56	0.16-1.01
p	0.086		**0.003***
**miR-181c**			
RQ median	0.09	0.07	0.23
Range	0.00-0.80	0.01-0.45	0.02-0.39
p	0.953		**0.009***
**miR-370**			
RQ median	0.33	0.22	0.45
Range	0.09-1.90	0.05-0.66	0.14-1.05
p	0.101		**0.018***
**miR-375**			
RQ median	0.12	0.12	0.34
Range	0.05-0.43	0.05-0.43	0.08-0.86
p	0.546		**0.001***
**miR-223**			
RQ median	0.27	0.71	0.62
Range	0.04-1.80	0.29-2.78	0.10-2.03
p	**0.009***		0.670

RQ = Relative Quantification; p: Mann-Whitney test or Wilcoxon matched pairs test;
* significant difference

Table S4: Comparisons between the relative expression of evaluated miRNAs according with *cagA* genotype in *H. pylori*-positive (Hp+) chronic gastritis patients before treatment.

Gene	*cagA*+		*cagA*-
**miR-103**			
RQ median	0.29		0.26
Range	0.16-0.49		0.14-0.33
p		0.691	
**miR-181c**			
RQ median	0.14		0.07
Range	0.03-0.45		0.02-0.19
p		0.548	
**miR-370**			
RQ median	0.18		0.21
Range	0.06-0.28		0.05-0.66
p		0.792	
**miR-375**			
RQ median	0.12		0.12
Range	0.07-0.43		0.07-0.23
p		1.000	
**miR-223**			
RQ median	0.81		1.12
Range	0.38-0.92		0.50-2.78
p		0.310	

RQ = Relative Quantification; p: Mann-Whitney test
