# Supplementary material for: Interaction between inflammatory mediators and miRNAs in Helicobacter pylori infection
Source: Cell Microbiol. 2016 Apr 14;18(10):1444–58. doi: 10.1111/cmi.12587 (PMC5074252; doi:10.1111/cmi.12587)
Supplement: Supplementary file 1 — Supporting info item [file CMI-18-1444-s001.zip › Supplemetary material.doc]

**Table S1:** Relative expression of *TNFA, IL6, IL1B, IL12A, IL2* and *TGFBRII* mRNA in *H. pylori*-negative (Hp-) chronic gastritis patients and *H. pylori*-positive (Hp+) chronic gastritis patients before and after eradication.

| **Genes** | **Hp-** | **Before Treatment (Hp+)** | | **After eradication (Hp-)** |
| --- | --- | --- | --- | --- |
| ***TNFA*** |  |  | |  |
| RQ median | 1.35 | 2.58 | | 1.44 |
| Range | 0.38-4.26 | 0.49-4.65 | | 0.54-3.07 |
| p | **0.024*** | | **0.008*** | |
| ***IL6*** |  |  | |  |
| RQ median | 0.74 | 7.39 | | 2.90 |
| Range | 0.15-4.81 | 0.37-32.02 | | 0.63-12,90 |
| p | **<0.001*** | | **0.050*** | |
| ***IL1B*** |  |  | |  |
| RQ median | 1.58 | 1.78 | | 2.40 |
| Range | 0.61-3.07 | 0.98-7.44 | | 0.93-8.98 |
| p | 0.569 | | 0.130 | |
| ***IL12A*** |  |  | |  |
| RQ median | 0.87 | 2.00 | | 1.98 |
| Range | 0.17-3.13 | 0.39-5.01 | | 0.67-9.53 |
| p | **0.015*** | | 0.384 | |
| ***IL2*** |  |  | |  |
| RQ median | 0.35 | 0.75 | | 1.04 |
| Range | 0.05-2.49 | 0.23-1.84 | | 0.21-11.56 |
| p | **0.046*** | | 0.107 | |
| ***TGFBRII*** |  |  | |  |
| RQ median | 0.67 | 0.75 | | 0.80 |
| Range | 0.28-1.82 | 0.32-1.50 | | 0.32-2.90 |
| p | 0.964 | | **0.029*** | |

RQ = Relative Quantification; p: Mann-Whitney test or Wilcoxon matched pairs test;

* significant difference

**Table S2:** Comparisons between the relative expression of evaluated genes according with *cagA* genotype in *H. pylori*-positive (Hp+) chronic gastritis patients before treatment.

| **Gene** | ***cagA*+** | ***cagA*-** |
| --- | --- | --- |
| ***TNFA*** |  |  |
| RQ median | 3.32 | 3.25 |
| Range | 1.91-4.49 | 2.25-4.65 |
| p | 0.833 | |
| ***IL6*** |  |  |
| RQ median | 12.24 | 10.92 |
| Range | 3.05-21.59 | 4.22-32.02 |
| p | 1.000 | |
| ***IL1B*** |  |  |
| RQ median | 1.44 | 2.84 |
| Range | 0.98-4.39 | 1.24-7.44 |
| p | 0.240 | |
| ***IL12A*** |  |  |
| RQ median | 2.10 | 2.27 |
| Range | 1.48-2.76 | 1.36-5.01 |
| p | 0.628 | |
| ***IL2*** |  |  |
| RQ median | 0.61 | 1.04 |
| Range | 0.28-1.02 | 0.30-1.84 |
| p | 0.310 | |
| ***TGFBRII*** |  |  |
| RQ median | 0.61 | 0.76 |
| Range | 0.32-1.50 | 0.42-1.19 |
| p | 0.295 | |

RQ = Relative Quantification; p: Mann-Whitney test

**Table S3:** Relative expression of miR-103, miR-181c, miR-370, miR-375 and miR-223 in *H. pylori*-negative (Hp-) chronic gastritis patients and *H. pylori*-positive (Hp+) chronic gastritis patients before and after eradication.

| **microRNA** | **Hp-** | **Before Treatment (Hp+)** | | **After eradication (Hp-)** |
| --- | --- | --- | --- | --- |
| **miR-103** |  |  | |  |
| RQ median | 0.46 | 0.33 | | 0.68 |
| Range | 0.10-2.13 | 0.07-0.56 | | 0.16-1.01 |
| p | 0.086 | | **0.003*** | |
| **miR-181c** |  |  | |  |
| RQ median | 0.09 | 0.07 | | 0.23 |
| Range | 0.00-0.80 | 0.01-0.45 | | 0.02-0.39 |
| p | 0.953 | | **0.009*** | |
| **miR-370** |  |  | |  |
| RQ median | 0.33 | 0.22 | | 0.45 |
| Range | 0.09-1.90 | 0.05-0.66 | | 0.14-1.05 |
| p | 0.101 | | **0.018*** | |
| **miR-375** |  |  | |  |
| RQ median | 0.12 | 0.12 | | 0.34 |
| Range | 0.05-0.43 | 0.05-0.43 | | 0.08-0.86 |
| p | 0.546 | | **0.001*** | |
| **miR-223** |  |  | |  |
| RQ median | 0.27 | 0.71 | | 0.62 |
| Range | 0.04-1.80 | 0.29-2.78 | | 0.10-2.03 |
| p | **0.009*** | | 0.670 | |

RQ = Relative Quantification; p: Mann-Whitney test or Wilcoxon matched pairs test;

* significant difference

**Table S4:** Comparisons between the relative expression of evaluated miRNAs according with *cagA* genotype in *H. pylori*-positive (Hp+) chronic gastritis patients before treatment.

| **Gene** | ***cagA*+** | ***cagA*-** |
| --- | --- | --- |
| **miR-103** |  |  |
| RQ median | 0.29 | 0.26 |
| Range | 0.16-0.49 | 0.14-0.33 |
| p | 0.691 | |
| **miR-181c** |  |  |
| RQ median | 0.14 | 0.07 |
| Range | 0.03-0.45 | 0.02-0.19 |
| p | 0.548 | |
| **miR-370** |  |  |
| RQ median | 0.18 | 0.21 |
| Range | 0.06-0.28 | 0.05-0.66 |
| p | 0.792 | |
| **miR-375** |  |  |
| RQ median | 0.12 | 0.12 |
| Range | 0.07-0.43 | 0.07-0.23 |
| p | 1.000 | |
| **miR-223** |  |  |
| RQ median | 0.81 | 1.12 |
| Range | 0.38-0.92 | 0.50-2.78 |
| p | 0.310 | |

RQ = Relative Quantification; p: Mann-Whitney test
